# Cost-effectiveness of Chlamydia Vaccination Programs for Young Women

**DOI:** 10.3201/eid2106.141270

**Published:** 2015-06

**Authors:** Kwame Owusu-Edusei, Harrell W. Chesson, Thomas L. Gift, Robert C. Brunham, Gail Bolan

**Affiliations:** Centers for Disease Control and Prevention, Atlanta, Georgia, USA (K. Owusu-Edusei Jr, H.W. Chesson, T.L. Gift, G. Bolan);; University of British Columbia, Vancouver, British Columbia, Canada (R.C. Brunham)

**Keywords:** chlamydia, Chlamydia trachomatis, bacteria, chlamydial infections, chlamydia vaccination, vaccine, vaccinations programs, young women, cost-effectiveness, annual screening

## Abstract

We explored potential cost-effectiveness of a chlamydia vaccine for young women in the United States by using a compartmental heterosexual transmission model. We tracked health outcomes (acute infections and sequelae measured in quality-adjusted life-years [QALYs]) and determined incremental cost-effectiveness ratios (ICERs) over a 50-year analytic horizon. We assessed vaccination of 14-year-old girls and catch-up vaccination for 15–24-year-old women in the context of an existing chlamydia screening program and assumed 2 prevaccination prevalences of 3.2% by main analysis and 3.7% by additional analysis. Estimated ICERs of vaccinating 14-year-old girls were $35,300/QALY by main analysis and $16,200/QALY by additional analysis compared with only screening. Catch-up vaccination for 15–24-year-old women resulted in estimated ICERs of $53,200/QALY by main analysis and $26,300/QALY by additional analysis. The ICER was most sensitive to prevaccination prevalence for women, followed by cost of vaccination, duration of vaccine-conferred immunity, and vaccine efficacy. Our results suggest that a successful chlamydia vaccine could be cost-effective.

## Abstract 2

A successful vaccine could be a cost-effective addition to current screening practices.

Chlamydia remains a major public health problem; there were ≈105.7 million new cases of this disease among adults 15–49 years of age worldwide in 2008 (*1*). In the United States, >1.4 million cases of chlamydial infections were reported to the Centers for Disease Control and Prevention in 2012 (*2*). A recent study estimated that there were ≈2.8 million cases of chlamydia among all persons of all ages in 2008 (*3*) and that the estimated direct lifetime cost was >$500 million 2013 US dollars (*4*). Most infections in women are asymptomatic, and untreated infections can progress to serious sequelae, such as pelvic inflammatory disease (PID), ectopic pregnancy, tubal infertility, and chronic pelvic pain (*5**,**6*). In addition, untreated chlamydia may cause serious and costly sequelae, such as urethritis, epididymitis, proctitis, and Reiter syndrome in men (*5*).

In this study, we explored the health and economic outcomes of a hypothetical chlamydia vaccine in the United States from a societal perspective. Although there currently is no chlamydia vaccine, the future development of an effective chlamydia vaccine is possible, and support for use of a vaccine for future chlamydia prevention efforts continues to increase (*7**–**10*). Models of the effect and cost-effectiveness of human papillomavirus (HPV) vaccine were developed before HPV vaccines were approved for use in the United States. These models, as well as subsequent models they helped to inform, proved valuable to public health officials and policy makers (*11**–**14*). Our exploratory model is intended to help advance the discussion surrounding development of a successful chlamydia vaccine, to inform the business case for investing in research and development of chlamydia vaccines, and to promote development of more detailed models so that the necessary tools are in place for chlamydia vaccine recommendations.

## Methods

### Model Summary

**Figure 1:**
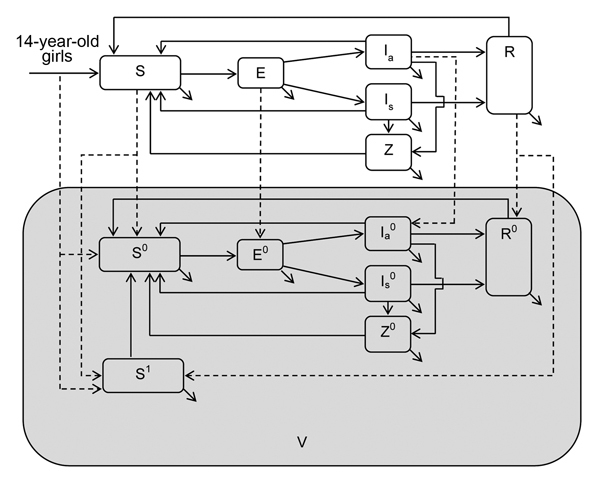
Schematic for exploring the cost-effectiveness of the hypothetical chlamydia vaccine. S, susceptible; E, exposed; I_a_, infectious asymptomatic; I_s_, infectious symptomatic; R, infection-conferred immunity; Z, sequelae; V (shaded area), vaccinated; superscripts, none, not vaccinated; 0, vaccinated but not effective; 1, vaccinated and effective. Infected persons move into the exposed (E, incubation compartment). From E, they move to either the infectious asymptomatic (Ia) or infectious symptomatic (Is) compartment on the basis of the probability of being symptomatic and the duration of incubation. Infected persons may recover naturally and move to the infection-conferred immunity compartment R, receive treatment, and move back to the susceptible compartment S or show development of chlamydia-associated complications and enter the sequelae compartment Z. Vaccinated persons enter the super compartment V and those whose vaccination was not effective continue to move through the health status compartment (S^0^, E^0^, Ia^0^, Is^0^, Z^0^, R^0^) states similar to those not vaccinated (S, E, Ia, Is, Z, R) because we assumed that there was no prophylactic benefit for that group. Conversely, persons with effective vaccination are not susceptible (S^1^). They stay immune for a specified time on the basis of the duration of the vaccine-conferred immunity applied, after which they move to the vaccinated but not effective compartment (S^0^). No vaccination was applied to persons in the infectious symptomatic and sequelae compartments. Also, the vaccine was assumed to be ineffective for persons in the exposed (E) and infectious asymptomatic (I_a_) compartments. Because we assumed that sexual debut (first sexual intercourse) is at the age of 15 years, 14-year-old girls enter the susceptible compartments (S, S^0^, and S^1^) on the basis of vaccination coverage and vaccine efficacy values applied.

Institutional review board approval was not required for this study because we used only secondary data. To assess the health and economic outcomes of a hypothetical chlamydia vaccine for young persons (15–24 years of age), we accounted for herd effects by using a heterosexual transmission model. We constructed a relatively simple deterministic population-based compartmental model of chlamydia transmission (Figure 1) on the basis of previously published models (*15**–**17*). We assumed a population of 100,000 (50% men and 50% women) (*13**,**16*). To simplify our model, our population was made up of 1 age group (men and women 15–24 years of age) that has the highest risk for chlamydia infection in the United States (*3*). Thus, our model was not age-structured.

Given that our model population consisted of 10 birth cohorts (ages 15–24 years), we assumed that annual entry and exit into the population of 15–24-year-old persons was ≈10% of the population. In addition, we assumed that the age at sexual debut (first sexual intercourse) for girls and boys was 15 years. Thus, 14-year-old persons who turned 15 entered the model in susceptible compartments, and 24-year-old persons who turned 25 exited the model at the end of each year, such that the total population was constant at any given time over the analytic horizon (Figure 1). We accounted for heterogeneity in sexual behavior by assuming 2 classes of sexual activity (high and low) on the basis of the annual number of new sex partners. Other details of the model and associated equations are provided in the online Technical Appendix (http://wwwnc.cdc.gov/EID/article/21/6/14-1270-Techapp1.pdf). We assembled data for the model from published reports (Table 1).

**Table 1:** Model parameters, base-case values, and ranges used in a model to assess health and economic outcomes of a hypothetical chlamydia vaccine*

Parameter	Value (range)		Reference
	Men	Women	
Duration of symptomatic infection, d	14 (10–21)	28 (10–35)	(*15**,**16*)
Duration of asymptomatic infection, d	182.5 (120–240)	365 (240–480)	(*15**,**16*)
Incubation period, d	14 (7–21)	14 (7–21)	(*15**,**16*)
Duration of sequelae, d	21 (10–30)	60 (45–75)	(*16*)
Probability of sequelae, %	2 (0–5)	15 (10–20)	(*16**,**18*)
Per-partnership transmission probability, %	70 (25–80)	68 (25–80)	(*19*)
Probability of symptomatic infection, %	50 (20–80)	20 (10–50)	(*15**,**16*)
Average no. partners in past year, high sexual activity	13.30 (10.00–16.00)	33.26 (30.00–40.00)	(*15**,**16**,**20*)
Average no. partners in past year, low sexual activity	0.90 (0.60–1.20)	0.88 (0.60–1.50)	(*15**,**16**,**20*)
Proportion in low sexual activity class, %	95.0 (90.0–99.0)	97.9 (95.0–99.0)	(*15**,**16**,**20*)
Annual screening rate, %	0	30 (10–50)	(*15*)
Probability of postscreening treatment, %	80 (50–99)	80 (50–99)	(*15*)
Probability of treatment, symptomatic, %	89 (80–100)	89 (80–100)	(*4*)
Test sensitivity, %	95 (90–100)	95 (90–100)	(*21*)
Test specificity, %	99 (95–100)	99 (95–100)	(*21*)
Treatment efficacy (doxycycline, azithromycin), %	92 (80–100)	92 (80–100)	(*15**,**22*)
QALYs lost/case			
Symptomatic infection	0.005646 ± 50%	0.009913 (± 50%)	(*16*)
Sequelae†	0.009530 ± 50%	0.497580 (± 50%)	(*16*)
Costs (2013 US Dollars)			
Treatment of acute chlamydia‡	185.2 ± 50%	183.0 (± 50%)	(*4**,**23**–**25*)
Sequelae†	1,337 ± 50%	4,516 (± 50%)	(*4**,**16**,**26*)
Screening	55 ± 50%	55 (± 50%)	(*4**,**23*)
Vaccination	547 ± 50%	547 (± 50%)	Model assumption
Vaccine coverage, 14-y-old persons, %	0	30 (10–50)	Model assumption (*27*)
Vaccine coverage, 15–24-y-old persons, %	0	30	Model assumption (*27*)
Vaccine efficacy, %	75 (50–100)	75 (50–100)	Model assumption (*27*)
Duration of vaccine-conferred immunity, y	10 (1–100)	10 (1–100)	Model assumption
Duration of infection-conferred immunity, y	1 (0.5–5.0)	1 (0.5–5.0)	(*17*)
Relative size of the 14-y-old population entering model compared with overall population model, %	10 (5–15)		Model assumption
Sexual mixing parameter§	0.50 (0.10–0.90)		Model assumption
Discount rate, %	3 (0–10)		Model assumption

*QALYs, quality-adjusted life years. †Includes productivity costs or QALYs (where applicable) for epididymitis for men and complications associated with pelvic inflammatory diseases (i.e., chronic pelvic pain, ectopic pregnancy, and infertility) for women. ‡Includes productivity costs associated with acute chlamydia and seeking treatment (*24*) and the reported youth (16–24-y-old persons) employment rate in 2010 (48.9%) (*25*). §Used to determine the degree of mixing between the 2 (high and low) sexual activity groups (0, random mixing; 1, fully assortative).

Preliminary analyses, as well as results from other cost-effectiveness studies, indicated that the burden of chlamydia was an influential variable. Thus, we conducted 2 analyses: main analysis and additional analysis. In the main analysis, parameter values were selected from within published ranges such that the resulting chlamydia prevalence for women in the model was near the US national average for women 15–24 years of age (i.e., 3.2%) (*3*) after accounting for the current screening rate of 30%. In the additional analysis, we modified the model by using parameter values from within published ranges of key parameters such that the resulting chlamydia prevalence for women was 0.5% higher than was used in main analysis (i.e., 3.7% and a screening rate of 30%). Specifically, this was achieved by changing the proportion of women and men in the low sexual activity group from 97.9% to 97.6% and from 95.0% to 95.5%, respectively. Essentially, we increased the proportion of women in the high sexual activity group by 0.3% and decreased the proportion of men in the high sexual activity group by 0.5%. These changes were made to provide more information on the resulting health and economic outcomes in a population with a higher chlamydia prevalence.

### Vaccine Characteristics

We assumed that vaccine efficacy was 75% at a cost of $547 (2013 US dollars, cost of complete vaccine series per person) and provided immunity for an average of 10 years. As has been performed in most published studies on vaccine cost-effectiveness (*8**,**13**,**14**,**28*), we repeated the analysis using 100% efficacy and lifelong duration of vaccine immunity. We assumed that the chlamydia vaccine was prophylactic; thus, there were no therapeutic benefits to recipients who were already exposed/infected. We also assumed that persons with symptomatic infections or sequelae were not vaccinated. On the basis of current coverage of HPV vaccine (*27*), we assumed that chlamydia vaccine coverage for girls 14 years of age and women 15–24 years of age would be 30% achieved by a linear increment during the first 5 years of the onset of the vaccination program and would remain at that rate over the analytic horizon.

### Evaluation of Strategies and Health Outcomes

The 4 strategies assessed were A) no screening, no vaccination; B) screening women 15–24 years of age; C) screening women 15–24 years of age and vaccinating girls 14 years of age; and D) screening women 15–24 years of age, vaccinating girls (14-year-old), and catch-up vaccination for women 15–24 years of age. Thus, all persons vaccinated were also subject to annual screening at the same rate as persons who were not vaccinated. For cost purposes, it was assumed that screening would be conducted opportunistically when patients sought other care. Therefore, no productivity costs were assessed for screening.

Health outcomes were measured in quality-adjusted life-years (QALYs) estimated by using health state utility weights for acute infections and sequelae for men (epididymitis) and women (PID), including chronic pelvic pain, ectopic pregnancy, and infertility (*16*). Cumulative cost and effects (QALYs) were estimated over a 50-year time frame and analytic horizon for all strategies. All outcomes (cost and effects) were discounted at an annual rate of 3%. All costs were adjusted to 2013 US dollars by using the Medical Care component of the Consumer Price Index (*29*). To provide summaries of cost-effectiveness results from a societal perspective, we included productivity costs in the cost of diseases.

### Sensitivity Analyses

We assessed the sensitivity of our results to numerous parameter values (n = 44) that we used in our model. Specifically, we first used the Latin hypercube sampling (*4**,**30*) method to create 120 random combinations of parameter values by randomly choosing (without replacement) from 120 equiprobable parameter value intervals from ranges provided in Table 1. To explore all values in specified ranges equally, we assumed uniform distribution for all variables. Next, we ran each simulation and checked to ensure that a steady-state was reached before and after introducing the strategy. We recorded the resulting prevalence (for men and women), costs, and QALYs before and after the vaccination program. We then ranked all values (i.e., parameter values, prevalence and incremental cost-effectiveness ratios [ICERs]) and determined the partial rank correlation coefficients (PRCCs). The PRCCs provided the magnitude of the effect of the referent parameter on the ICER after partially eliminating effects of the other parameters.

In preliminary analyses, we found that prevaccination steady-state prevalence could vary substantially in the sensitivity analyses and that prevaccination prevalence for women was an influential determinant of the effect and cost-effectiveness of the vaccine program. Thus, we divided the PRCC analyses into 2 parts. In the first part, we determined the causal parameters for the prevaccination prevalence and then excluded these parameters from the second and final PRCC analysis, in which we determined the influential variables/parameters of the ICER. Thus, we determined the influential parameters of the prevaccination prevalence for women and included the prevaccination prevalence for women in the second and final PRCC analysis to determine the influential variables/parameters of the ICER. For the sensitivity analyses, we focused on the ICER for strategy C (screen women 15–24 years of age and vaccinate girls 14 years of age) when compared with strategy B (screen women 15–24 years of age).

## Results

### Main Analysis

**Figure 2:**
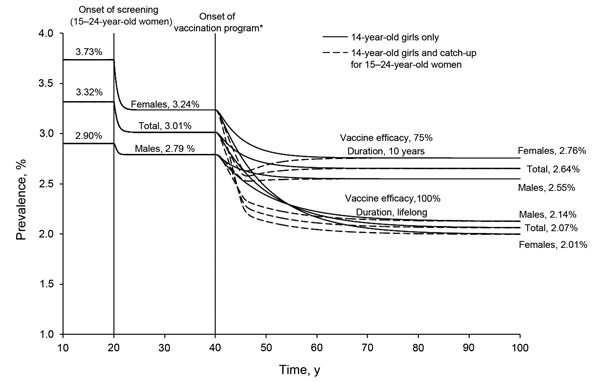
Time-prevalence chart for annual screening for 15–24-year-old women and a hypothetical chlamydia vaccine program for preadolescent girls (14 years of age) and women 15–24 years of age in the United States from the main analyses. We separated the start of the different programs (i.e., screening and vaccination) for illustrative purposes and to avoid clutter. When estimating the health and economic outcomes, we assumed that the strategy being analyzed started at the 20-year mark and the outcomes were tracked over a 50-year period (analytic horizon) ending at the 70-year mark. *Includes the existing annual screening (15–24-year-old women) strategy. Screening and vaccination coverage were 30% for all applicable age groups.

In the base-case scenario, chlamydia prevalence in the strategy A scenario (no screening, no vaccination) was 3.73% in women and 2.90% in men. With annual chlamydia screening coverage of 30% (the approximate status quo in the United States), chlamydia prevalence decreased from 3.73% to 3.24% for women and from 2.90% to 2.79% for men (Figure 2). The estimated ICER of strategy B (screen women 15–24 years of age) when compared with strategy A (no screening, no vaccination) was $38,700/QALY gained (Table 2). When vaccinating 14-year-old girls only in addition to screening (i.e., strategy C: screen women 15–24 years of age and vaccinate girls 14 years of age), the chlamydia prevalence was reduced to 2.76% for women and to 2.55% for men, and the estimated ICER of vaccination when compared with the status quo strategy B (i.e., screening 15–24-year-old women) was $35,300/QALY gained (Table 2).

**Table 2:** Summary health and cost outcomes for a hypothetical population of 100,000 persons for the examined interventions strategies for the main analysis (3.2% chlamydia prevalence for women 15–24 years of age)*

Strategy	Cumulative sequelae		Total cost†	QALYs lost	Incremental		
	Men	Women			Cost†	QALYs	$/QALY
A) No screening, no vaccination	1,654	7,458	54,159,500	4,268	Referent	Referent	Referent
B) Screening 15–24-year-old persons	1,593	6,515	72,823,100	3,786	18,663,600	482	38,700
75% efficacy lasting an average of 10 years							
C) Screening 15–24-year-old persons and vaccinating 14-year-old persons	1,487	5,767	87,480,600	3,371	14,657,600‡	415‡	35,300
D) Screening 15–24-year-old persons, vaccinating 14-year-old persons, and catch-up vaccination of 15–24-year-old persons	1,466	5,558	93,540,000	3,257	6,059,300	114	53,200
100% efficacy lasting for life							
Repeat C	1,352	4,903	81,495,900	2,889	8,672,800‡	897‡	9,700
Repeat D	1,297	4,423	85,773,100	2,624	4,277,200	265	16,100

*All outcomes (cumulative sequelae, quality-adjusted life-years [QALYs], and costs) have been discounted at an annual rate of 3%. †Costs are in 2013 US dollars and rounded to the nearest hundred. ‡Incremental cost and QALYs when compared with strategy B (screening 15–24-year-old persons). Although this strategy was weakly dominated, we did not eliminate it because we wanted to show how the vaccine strategies compared with the status quo or existing strategy (B).

Including catch-up vaccination for 15–24-year-old women (i.e., strategy D, screen women 15–24 years of age, vaccinate girls 14 years of age, and catch-up vaccination for women 15–24 years of age) did not change the long-term reduction in chlamydia prevalence relative to strategy C (Figure 2). However, reductions in chlamydia prevalence were achieved more rapidly than without catch-up vaccination (Figure 2). The estimated ICER of adding catch-up vaccination when compared with strategy C (screen women 15–24 years of age and vaccinate girls 14 years of age) was $53,200/QALY gained. Throughout the analyses, although strategy B was weakly dominated, we did not eliminate it because we wanted to show how vaccine strategies compared with the status quo or existing strategy B (screen females 15–24 years of age).

When we applied values for perfect vaccine performance (i.e., 100% efficacy and lifelong duration of immunity), the chlamydia prevalence in strategy C (screen women 15–24 years of age and vaccinate girls 14 years of age) was reduced further, to 2.01% for women and to 2.14% for men (Figure 2), and the ICER when compared with strategy B (screen women 15–24 years of age) was reduced to $9,700/QALY gained. Adding catch-up vaccination for 15–24 year-old women (i.e., strategy D: screen women 15–24 years of age, vaccinate girls 14 years of age, and catch-up vaccination for women 15–24 years of age) compared with strategy C (screen women 15–24 years of age and vaccinate girls 14 years of age) had an ICER of $16,100/QALY gained (Table 2).

When we assumed perfect chlamydia vaccine performance (i.e., 100% efficacy and lifelong duration of immunity) and increased coverage for 14-year-old persons to >75%, our results indicated that overall illness from chlamydia decreased by ≈ 90% in 20 years. In addition, illness from chlamydia was eliminated in ≈30 years after onset of the vaccination program.

### Additional Analysis

Results for additional analysis were similar in relative terms to what we found for main analysis. However, because of higher chlamydia prevalence in additional analysis, the estimated ICERs were substantially lower (<50%) than we found for main analysis (Table 3). When we applied values for perfect vaccine performance (i.e., 100% efficacy and lifelong duration of immunity), the estimated ICER for strategy C (screen women 15–24 years of age and vaccinate girls 14 years of age) was cost-saving (Table 3). Adding a catch-up vaccination program for 15–24-year-old women (i.e., strategy D: screen women 15–24 years of age, vaccinate girls 14 years of age, and catch-up vaccination for women 15–24 years of age) was also highly cost-effective ($1,500/QALY gained over strategy C [screen women 15–24 years of age and vaccinate girls 14-years of age]).

**Table 3:** Summary health and cost outcomes for a hypothetical population of 100,000 persons for the examined interventions strategies for the additional analysis (3.7% chlamydia prevalence for women 15–24 years of age)*

Strategy	Cumulative sequelae		Total cost†	QALYs lost	Incremental		
	Men	Women			Cost†	QALYs	$/QALY
A) No screening, no vaccination	1,720	8,610	63,744,600	5,161	Referent	Referent	Referent
B) Screening 15–24-year-old persons	1,635	7,465	82,743,300	4,282	18,998,700	879	21,600
75% efficacy lasting an average of 10 years							
C) Screening 15–24-year-old persons and vaccinating 14-year-old persons	1,568	6,931	87,498,800	3,989	4,755,500‡	293‡	16,200
D) Screening 15–24-year-old persons, vaccinating 14-year-old persons, and catch-up vaccination of 15–24-year-old persons	1,540	6,629	91,820,000	3,825	4,321,200	164	26,300
100% efficacy lasting for life							
Repeat C	1,457	6,122	82,059,500	3,541	−683,800‡	741‡	Cost-saving
Repeat D	1,368	5,252	82,750,200	3,067	690,700	474	1,500

*All outcomes (cumulative sequelae, quality-adjusted life-years [QALYs], and costs) have been discounted at an annual rate of 3%. †Costs are in 2013 US dollars and rounded to the nearest hundred. ‡Incremental cost and QALYs when compared with strategy B (screening 15–24-year-old persons). Although this strategy was weakly dominated, we did not eliminate it because we wanted to show how the vaccine strategies compared with the status quo or existing strategy (B).

### Sensitivity Analyses

A summary of results from the first part of the PRCC analyses used to determine the hierarchy of influential parameters for preintervention prevalence in women is shown in Table 4. Our results indicated that the preintervention prevalence for women was highly sensitive to the proportion of women in the low (or high) sexual activity category, followed by the duration of infection-conferred immunity, per-partner transmission probability (man to woman), duration of asymptomatic infections (woman followed by man), mixing parameter, probability of symptomatic infection (woman followed by man), annual screening coverage (women), number of partners in the past year for women with low sexual activity, number of partners in the past year for women with high sexual activity, number of partners in the past year for men with low sexual activity, duration of symptomatic infections in women, probability of postscreening treatment, and relative size of the population of persons 14 years of age entering the model each year.

**Table 4:** Summary rank regression results for select parameters used in the model to determine the health and economic outcomes of a hypothetical chlamydia vaccine

Variable/parameter*	Rank coefficient†	p value
Dependent variable: prevaccination prevalence in women		
Proportion of women in low activity class	−0.85	0.0001
Duration of infection-conferred immunity	−0.77	0.0001
Per-partner probability of transmission, man to women	0.73	0.0001
Duration of asymptomatic infection in women	0.50	0.0001
Duration of asymptomatic infection in men	0.49	0.0001
Mixing parameter	−0.45	0.0001
Proportion of symptomatic infections for women	−0.40	0.0001
Proportion of symptomatic infections for men	−0.38	0.0001
Annual screening coverage for women	−0.36	0.0001
No. partners in past year, low sexual activity women	0.30	0.0001
No. partners in past year, high sexual activity women	0.30	0.012
No. partners in past year, low sexual activity men	0.27	0.013
Duration of symptomatic infection for women	0.21	0.047
Probability of postscreening treatment	−0.18	0.069
Relative size of the 14-y-old population	0.12	0.091
Dependent variable: incremental cost-effectiveness ratio		
Prevaccination prevalence for women	−0.77	0.0001
Vaccine cost	0.71	0.0001
Duration of vaccine-conferred immunity	−0.50	0.0001
Vaccine efficacy	−0.45	0.0001
Probability of sequelae for women	−0.32	0.0001
Discount rate	0.29	0.0001

*Only variables/parameters for which p<0.10 are shown. †Presented in decreasing order of absolute magnitude.

The second and final part of the PRCC analyses used to determine the hierarchy of influential parameters/variables of the ICER is shown in Table 4. Our results showed that the most influential variable on the estimated ICER was the prevaccination prevalence in women, followed by 3 vaccine-related variables (vaccine cost, duration of vaccine-conferred immunity, and vaccine efficacy), probability of sequelae in women, and the discount rate.

**Figure 3:**
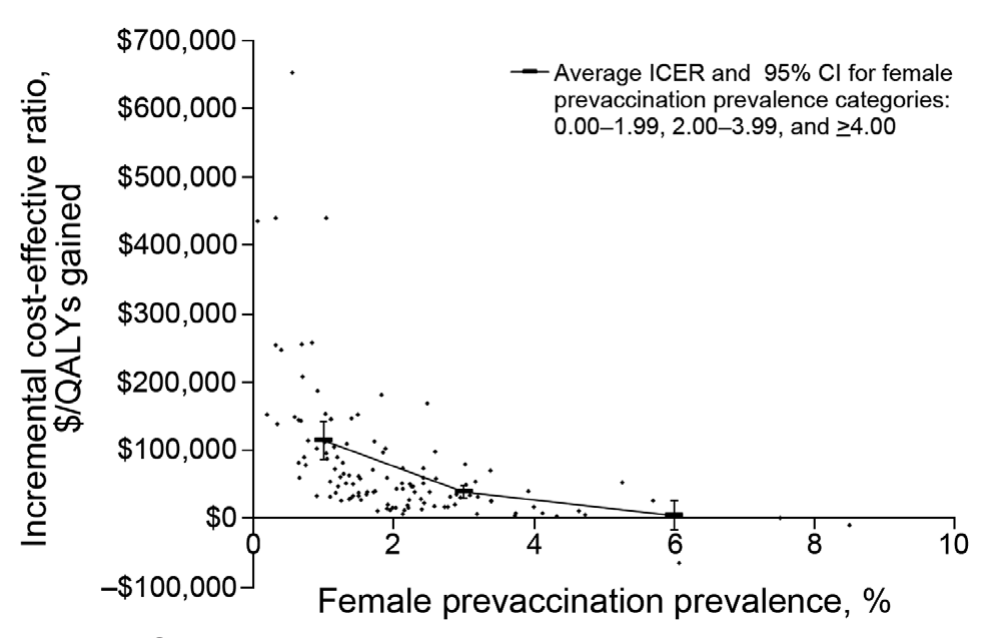
Sensitivity analyses (scatter diagram) showing incremental cost-effectiveness ratios (ICERs) versus female prevaccination prevalence for a hypothetical chlamydia vaccine program. QALYs, quality-adjusted life-years.

The estimated prevaccination prevalence for women ranged from 0.06% to 8.51% (mean 2.06%, 95% CI 1.81%–2.31%). The overall average ICER was $86,349/QALY gained (95% CI $66,910–$105,789), but this value was largely attributable to scenarios with low prevalence of chlamydia. When looking at the ICERs for female prevaccination prevalence cutoffs (0.00–1.99, 2.00–3.99, and >4.00), the average ICERs were $125,087/QALY gained (95% CI $94,422–$155,752), $43,037/QALY gained (95% CI $32,824–$53,248), and $4,849/QALY gained (95% CI cost-saving–$28,344], respectively (Figure 3). When prevaccination prevalence for women was 2%–3%, the estimated average ICER was $44,486/QALY gained (95% CI $31,772–$57,202). Finally, when we limited the analysis to include only parameter sets that resulted in chlamydia prevalence within the CIs reported for chlamydia prevalence in the United States (i.e., 2.26%–4.52%) (*3*), the estimated average ICER was $42,378/QALY gained (95% CI $29,619–$55,136).

## Discussion

We used a deterministic heterosexual transmission model that was relatively simple compared with previously published models (*11**–**14**,**20**,**31*) to explore the potential health and economic outcomes of a hypothetical chlamydia vaccine focusing on vaccination programs for 14-year-old girls and 15–24-year-old women in the United States. We repeated our analyses by using a higher disease burden. Overall, results from our exploratory analyses showed that a chlamydia vaccine could be cost-effective under many plausible scenarios. Interventions that reduce QALYs lost for <1–3 times per capita gross domestic product (≈$50,000 in the United States) are typically considered to be cost-effective (*32*). Our sensitivity analyses suggest that a highly efficacious chlamydia vaccine with long duration of immunity might be cost-saving in countries with high prevalence of chlamydia, as demonstrated by results of our additional analysis. Our results are consistent with preliminary, spreadsheet-based calculations, which suggested that a chlamydia vaccine would cost <$10,000/QALY saved (*28*).

Our analyses showed that a high-performance vaccine could potentially eliminate chlamydia infection when coverage was high (>75%) among susceptible persons before their sexual debut. These results were consistent with findings from previous studies (*17**,**33*), and our estimates of cost-effectiveness of chlamydia screening (versus no screening) were consistent with those of previous studies (*16**,**34*). In addition, the relative cost-effectiveness of targeting different age groups was consistent with results of previous studies on HPV vaccine (*11**–**14*). In particular, our study showed that catch-up vaccination of 15–24-year-old women, in addition to 14-year-old girls, resulted in an increase in the ICER, implying that additional QALYs are gained at higher costs. Consistent with results of Elbasha et al. (*13*) the addition of catch-up vaccination of 15–24-year-old women did not change the long-term prevalence of infection, but did shorten the time needed to realize the effects of vaccination.

An additional aspect of vaccination is that it is easier to implement than an intervention of routine screening because it does not need to be repeated annually. Although health services data have shown chlamydia screening rates >30% in young women (*35*), time-series insurance data have shown that <1% of women <25 years of age are consistently screened at least once per year (*36*).

Our exploratory study has several limitations. Notable among them is the inherent limitations associated with models in general because models are simplifications of real-world events. Thus, all limitations associated with models are applicable. Another major limitation is the high uncertainty surrounding the parameter values we used (including illness estimates). Because we focused on heterosexual transmission, our model was largely driven by parameters associated with women; prevaccination prevalence was calibrated to approximate reported illnesses for women, and prevaccination prevalence for men was determined by the model. Because a substantially high proportion of high-impact health outcomes of chlamydia infection are in women (i.e., PID and associated complications), it is reasonable to focus on illness in women in such analyses. Nonetheless, as was conducted for HPV (*11**,**13*), future studies should also assess cost-effectiveness of chlamydia vaccination for men.

The prevaccination prevalence rates for men determined by our model were substantially different from reported prevalence rates for men in the United States. For instance, the reported prevalence for men of a similar age group (15–24 years) in the United States was approximately half that of women (men 1.66%; women 3.21%) (*3*), and prevalence in men from our main analysis was substantially higher (men 2.79%; women 3.24%).

We excluded numerous possible outcomes of chlamydia vaccination, such as changes in the number of partners or screening practices, which might arise as a result of vaccination, health benefits for persons vaccinated while infected, and costs and loss in quality of life to persons who experience potential adverse vaccination outcomes, such as side effects (e.g., temporary pain at injection site) (*11*).

We did not explore potential broader properties of an effective chlamydia vaccine, such as degree (i.e., reducing susceptibility but not completely eliminating it) or infectiousness (i.e., breakthrough infections being less infectious than primary infections and shorter in duration). Future studies should consider assessing these 2 characteristics (degree and infectiousness). We assumed that all members of the hypothetical population (with substantially different sexual activity levels) have equal access to screening, treatment, and vaccination. Thus, treatment rate, screening rate, and vaccination coverage were applied equally across all eligible model compartments (subpopulations). However, this simplifying assumption is probably not realistic. Consequently, benefits of screening and vaccination might have been overestimated if women who are highly sexually active were less likely to be screened, treated, or vaccinated. In addition, it is also conceivable that persons vaccinated might be less likely to be screened for chlamydia annually. Further studies are needed to explore the potential health and economic benefits of a chlamydia vaccine that targets specific subpopulations, such as persons infected, those with limited access to health care, and those who have multiple sexual partners.

Because our model does not account for major factors, such as age-based mixing of sexual partners and ongoing sexual partnerships, our model is not of sufficient complexity to inform chlamydia vaccine recommendations. For example, our model assumed sexual debut at 15 years of age and that sex partners were chosen from a pool of 15–24 year-old persons, thereby ignoring heterogeneity in age at sexual debut, which is a simplification (*37*). Similarly, models such as ours that do not specifically keep track of ongoing sexual partnerships can overestimate the effect of chlamydia screening because reinfection of treated women by their untreated sex partner is not specifically taken into account (*38**,**39*). If the effect of chlamydia screening is overestimated, then the marginal effect of adding chlamydia vaccination to an existing chlamydia screening program might be underestimated. Development of more complex models will be needed over time, and these models would be better suited to examine the effect of vaccination over a wide range of assumptions regarding vaccine coverage, efficacy, and duration of protection.

Notwithstanding these limitations, our model provides useful information on the potential cost-effectiveness of a chlamydia vaccine, as well as a useful basis for future chlamydia vaccine cost-effectiveness analyses and other modeling studies. In particular, determination of the hierarchy of influential parameters in our model would be useful for future analyses, and assist in understanding the relative roles played by numerous variables that are used in models to facilitate discussions around simple and complex model inputs. Finally, our study suggests that a successful chlamydia vaccine could have a substantial effect on chlamydia prevalence, thereby reducing the health and economic burden associated with chlamydia.

**Technical Appendix.** Other details of a model and associated equations for a hypothetical chlamydia vaccine.
