# Supplementary material for: Cost-effectiveness of Chlamydia Vaccination Programs for Young Women
Source: Emerg Infect Dis. 2015 Jun;21(6):960–8. doi: 10.3201/eid2106.141270 (PMC4451885; doi:10.3201/eid2106.141270)
Supplement: Supplementary file 1 — Technical Appendix. Other details of a model and associated equations for a hypothetical chlamydia vaccine. [file 14-1270-Techapp-s1.pdf]

Article DOI: http://dx.doi.org/10.3201/eid2106.141270

Cost-effectiveness of Chlamydia Vaccination Programs for Young Women

Technical Appendix

Model Equations

Not Vaccinated

$$\frac{d}{dt}S_{ij} = -\gamma_{ij}S_{ij} + t_i qs_{ij} Is_{ij} + (1-v_{14i})\varphi N_{ij} - \varphi S_{ij} + \alpha_i (Ia_{ij} + Is_{ij}) + nR_{ij} + \delta_i Z_{ij} - v_{15-24i} S_{ij}$$

$$\frac{d}{dt}E_{ij} = \gamma_{ij}S_{ij} - \omega E_{ij} - \varphi E_{ij} - v_{15-24i} E_{ij}$$

$$\frac{d}{dt}Is_{ij} = \xi_i \omega E_{ij} - qs_i Is_{ij} - \varphi Ia_{ij} - \alpha_i Is_{ij}$$

$$\frac{d}{dt}Ia_{ij} = \omega E_{ij}(1-\xi_i) - qa_i Ia_{ij} - \varphi Ia_{ij} - \alpha_i Ia_{ij} - v_{15-24_i} Ia_{ij}$$

$$\frac{d}{dt}R_{ij} = (1-p_i)(1-t_i)qs_i Is_{ij} + (1-p_i)qa_i Ia_{ij} - \varphi R_{ij} - nR_{ij} - v_{15-24_i} R_{ij}$$

$$\frac{d}{dt}Z_{ij} = p_i(1-t_i)qs_i Is_{ij} + p_i qa_i Ia_{ij} - \delta_i Z_{ij} - \varphi Z_{ij}$$

Vaccinated (Not Effective)

$$\frac{d}{dt}S^0_{ij} = -\gamma_{ij}S^0_{ij} + t_i qs_i Is^0_{ij} - \varphi S_{ij} + \alpha_i (Ia^0_{ij} + Is^0_{ij}) + nR^0_{ij} + \delta_i Z^0_{ij} + (1-e)v_{15-24i} S_{ij} + (1-e)v_{14i}\varphi N_{ij} + mS^1_{ij}$$

$$\frac{d}{dt}E^0_{ij} = \gamma_{ij}S^0_{ij} - \omega E^0_{ij} - \varphi E^0_{ij} + v_{15-24i} E_{ij}$$

$$\frac{d}{dt}Is^0_{ij} = \xi_i \omega E^0_{ij} - qs_i Is^0_{ij} - \varphi Ia^0_{ij} - \alpha_i Is^0_{ij}$$

$$\frac{d}{dt}Ia^0_{ij} = \omega E^0_{ij}(1-\xi_i) - qa_i Ia^0_{ij} - \varphi Ia^0_{ij} - \alpha_i Ia^0_{ij} + v_{15-24_i} Ia_{ij}$$

$$\frac{d}{dt}R^0_{ij} = (1-p_i)(1-t_i)qs_i Is^0_{ij} + (1-p_i)qa_i Ia^0_{ij} - \varphi R^0_{ij} - nR^0_{ij} + (1-e)v_{15-24_i} R_{ij}$$

$$\frac{d}{dt}Z^0_{ij} = p_i(1-t_i)qs_i Is^0_{ij} + p_i qa_i Ia^0_{ij} - \delta_i Z^0_{ij} - \varphi Z^0_{ij}$$

Vaccinated (Effective)

$$\frac{d}{dt}S_{ij}^{1} = ev_{14i}\varphi N_{ij} + ev_{15-24i}(S_{ij} + R_{ij}) - \varphi S_{ij}^{1} - mS_{ij}^{1}$$

Population Size

$$N_{ij} = S_{ij} + E_{ij} + Is_{ij} + Ia_{ij} + R_{ij} + Z_{ij} + S_{ij}^{0} + E_{ij}^{0} + Is_{ij}^{0} + Ia_{ij}^{0} + R_{ij}^{0} + Z_{ij}^{0} + S_{ij}^{1}$$

Prevalence

$$\kappa_{ij} = \frac{Is_{ij} + Ia_{ij} + Is_{ij}^{0} + Ia_{ij}^{0}}{N_{ij}}$$

Total Infections

$$I_{ij} = Is_{ij} + Ia_{ij} + Is_{ij}^{0} + Ia_{ij}^{0}$$

Mixing Equation

$$\tau_{ijk} = \varepsilon M_{jk} + (1-\varepsilon)\left(\frac{c_{i'k}N_{i'k}}{\sum_{k} c_{i'k}N_{i'k}}\right)$$

Force of Infection

$$\gamma_{ij} = \beta_i c_{ij} \sum_{k} \tau_{ijk} \frac{I_{i'k}}{N_{i'k}}$$

Total discounted quality-adjusted life-years (QALYs) =

$$\int_{y=1}^{50} \frac{1}{\exp^{ry}}\left(qaly\_ct_i \xi_i \omega_i (E_{ij}^{0} + E_{ij}) + qaly\_seq_i (p_i (1-t) qs_i (Is_{ij} + Is_{ij}^{0}) + p_i qa_i (Ia_{ij} + Ia_{ij}^{0}))\right) \bullet dy$$

Total discounted cost =

$$\int_{y=1}^{50} \frac{1}{\exp^{ry}} \left(\begin{array}{l} vac\_cost \bullet v_{15-24}(S_{ij} + E_{ij} + Ia_{ij} + R_{ij}) + vac\_cost \bullet v_{14} \varphi N_{ij} \\ + s_i test\_cost_i N_{ij} \\ + p\_rx(test\_cost_i + rx\_cost_i) qs_i (Is_{ij} + Is_{ij}^0) \\ + s_i sens \bullet p\_rx\_sc \bullet rx\_cost_i (Ia_{ij} + Is_{ij} + Ia_{ij}^0 + Is_{ij}^0) \\ + s_i (1 - spec) p\_rx\_sc \bullet rx\_cost_i (S_{ij} + E_{ij} + R_{ij} + Z_{ij} + S_{ij}^0 + E_{ij}^0 + R_{ij}^0 + Z_{ij}^0 + S_{ij}^1) \\ + seq\_cost_i p_i (1 - t) qs_i (Is_{ij} + Is_{ij}^0) \\ + seq\_cost_i p_i qa_i (Ia_{ij} + Ia_{ij}^0) \end{array} \right) \bullet dy$$

Total discounted infections = $\int_{y=1}^{50} \frac{1}{\exp^{ry}} \left(\omega_i (E_{ij}^0 + E_{ij}) \right) \bullet dy$

Total discounted sequelae = $\int_{y=1}^{50} \frac{1}{\exp^{ry}} \left(p_i (1 - t) qs_i (Is_{ij} + Is_{ij}^0) + p_i qa_i (Ia_{ij} + Ia_{ij}^0) \right) \bullet dy$

Where S (susceptible), E (exposed), Is (infectious and symptomatic) and Ia (infectious and asymptomatic), R (infection-conferred immunity), and Z (sequelae) are the 6 compartments representing 6 mutually exclusive health status. Superscripts denote vaccine status and efficacy (none, not vaccinated; 0, vaccinated and not effective; 1 vaccinated and effective); subscripts i, and j represent sex (i = 1 for men, i = 2 for women) and sexual activity class (j = 1 for low, j = 2 for high), respectively, unless otherwise described. Rate of exit and entry into the population per year is represented by φ; the recovery rate is represented by qs/qa_i (qs for symptomatic infections; qa for asymptomatic infections); proportion treated successfully is represented by t (the product of probability of treatment [p_rx/p_rx_sc] and treatment efficacy [rx_success]); α is the annual screen-and-treat coverage, which is the product of the screening rate (s), test sensitivity (sens), postscreening treatment rate (p_rx_sc), and treatment efficacy (rx_sucess); n and m are the waning rates for infection-conferred and vaccine-conferred immunity, respectively; p is the probability of sequelae; δ represents the movement from sequelae to susceptible; e denotes vaccine efficacy; v_{14} and $v_{15–24}$ represent vaccine coverage for 14-year-old persons and 15–24-year-old persons, respectively; the proportion of symptomatic infections is ξ; rate of exit from the exposed state to the infectious states is ω (for simplicity we assumed the time

from infection to infectiousness is the same as the time from infection to symptoms); the force of infection (γ) is given by the product of the per-partner transmission probability (β), the rate of sex partner change (c), and the proportion of sex partners infected: determined by the mixing matrix ($\tau_{ijk,}$ where, subscript k is the sexual activity class of the partner) and the prevalence in the associated sexual-activity classes (κ); opposite subpopulation is differentiated by an apostrophe ('); M_{jk} represents full assortative mixing (equals 1 when j = k and 0 when $j \neq k$). Thus, when $\varepsilon = 0$, mixing is random and when $\varepsilon = 1$, mixing is fully assortative (*1*). Partnerships were balanced by adjusting the partnership rates using the relationship $c_{11}N_{11} = c_{21}N_{21}$ and $c_{12}N_{12} = c_{22}N_{22}$ with the assumption that women made the choice of partnership; partnerships by men were adjusted to equate partnerships by women (*1*,*2*). The discount rate is represented by r, y represents year, and exp is the transcendental number (2.71828).

We focused on a hypothetical population 15–24 years of age and assigned the respective lifetime costs and QALYs for each infection (and sequelae) on the basis of the published probabilities. In addition, the duration of immunity (vaccine-conferred and infection-conferred) were applied as rate of movement (inverse of duration) from the respective compartments. Thus, we did not explicitly track the health and economic outcomes (including the duration of vaccine protection) for those persons >24 years of age.

Additional Analysis

The model used for the additional analysis was the same as that used for the main analysis except for a few parameter values. Specifically, we decreased the proportion of women in the low sexual activity group from 97.9% to 97.6% and increased the proportion of men in the low sexual activity group from 95.0% to 95.5%. Essentially, the proportion of women in the high sexual activity group was increased by 0.3%, and the proportion of men in the high sexual activity group was decreased by 0.5%. The value of all other parameters, including costs remained the same as those used for the main analyses.

We used Berkeley Madonna version 8.3.9 (Robert I. Macey and George F. Oster, Berkeley, CA, USA) to solve the system of differential equations. We used an integration fixed time step size of 0.01 year (i.e., ≈4 days) and approximated the system of differential equations by using Runge-Kutta methods. The results were consistent when we repeated the analyses by using a shorter fixed time step of 0.001 year. We used Microsoft Excel version 2010 (Microsoft, Redmond, WA, USA) for creating the Latin hypercube sampling table for the sensitivity and uncertainty analyses. Finally, Stata version 11.1 (StataCorp LP, College Station, TX, USA) was used to conduct the partial rank correlation coefficient analyses.

Technical Appendix Table. Parameter values, ranges, and symbols/names used in the model of chlamydial vaccination*

Parameter	Value (range)		Symbol/parameter name
	Men	Women	
Duration of symptomatic infection, d	14 (10–21)	28 (10–35)	1/qs
Duration of asymptomatic infection, d	182.5 (120–240)	365 (240–480)	1/qa
Incubation period, d	14 (7–21)	14 (7–21)	$1/\omega$
Duration of sequelae, d	21 (10–30)	60 (45–75)	$1/\delta$
Probability of sequelae, %	2 (0–5)	15 (10–20)	p
Per-partnership transmission probability, %	70 (25–80)	68 (25–80)	β
Probability of symptomatic infection, %	50 (20–80)	20 (10–50)	ξ
Average no. partners in past year, high sexual activity	13.30 (10.00–16.00)	33.26 (30.00–40.00)	c
Average no. partners in past year, low sexual activity	0.90 (0.60–1.20)	0.88 (0.60–1.50)	c
Proportion in low sexual-activity class, %	95.0 (90.0–99.0)	97.9 (95.0–99.0)	p_low
Annual screening rate, %	0	30 (10–50)	s
Probability of postscreening treatment, %	80 (50–99)	80 (50–99)	p_rx_sc
Probability of treatment, symptomatic, %	89 (80–100)	89 (80–100)	p_rx
Test sensitivity, %	95 (90–100)	95 (90–100)	sens
Test specificity, %	99 (95–100)	99 (95–100)	spec
Treatment efficacy (doxycycline, azithromycin), %	92 (80–100)	92 (80–100)	rx_success
QALYs lost/case			
Symptomatic infection	0.005646 ± 50%	0.009913 (± 50%)	qaly_ct
Sequelae†	0.009530 ± 50%	0.497580 (± 50%)	qaly_seq
Costs (2013 US Dollars)			
Treatment of acute chlamydia‡	185.2 ± 50%	183.0 (± 50%)	rx_cost
Sequelae†	1,337 ± 50%	4,516 (± 50%)	seq_cost
Screening	55 ± 50%	55 (± 50%)	test_cost
Vaccination	547 ± 50%	547 (± 50%)	vac_cost
Vaccine coverage, 14-y-old persons, %	0	30 (10–50)	v_{14}
Vaccine coverage, 15–24-y-old persons, %	0	30	$v_{15–24}$
Vaccine efficacy, %	75 (50–100)	75 (50–100)	E
Duration of vaccine-conferred immunity, y	10 (1–100)	10 (1–100)	1/m
Duration of infection-conferred immunity, y	1 (0.5–5.0)	1 (0.5–5.0)	1/n
Relative size of the 14-y-old population entering model compared with the overall population in model, %	10 (5–15)		φ
Sexual mixing parameter§	0.50 (0.10–0.90)		ε
Discount rate, %	3 (0–10)		r

*QALYs, quality-adjusted life years.
†Includes productivity costs or QALYs (where applicable) for epididymitis for men and complications associated with pelvic inflammatory diseases (i.e., chronic pelvic pain, ectopic pregnancy, and infertility) for women.
‡Includes productivity costs associated with acute chlamydia and seeking treatment (*3*) and the reported youth (16–24-y-old persons) employment rate in 2010 (48.9% (*4*).
§Used to determine the degree of mixing between the 2 (high and low) sexual activity groups (0, random mixing; 1, fully assortative).

References

1. Garnett GP, Mertz KJ, Finelli L, Levine WC, St Louis ME. The transmission dynamics of gonorrhoea: modelling the reported behaviour of infected patients from Newark, New Jersey. Philos Trans R Soc Lond B Biol Sci. 1999;354:787–97. PubMed http://dx.doi.org/10.1098/rstb.1999.0431

2. Chen MI, Ghani AC, Edmunds WJ. A metapopulation modelling framework for gonorrhoea and other sexually transmitted infections in heterosexual populations. J R Soc Interface. 2009;6:775–91. PubMed

3. Owusu-Edusei K, Roby TM, Chesson HW, Gift TL. Productivity costs of nonviral sexually transmissible infections among patients who miss work to seek medical care: evidence from claims data. Sex Health. 2013;10:434–7. PubMed http://dx.doi.org/10.1071/SH13021

4. Bureau of Labor Statistics. The editor's desk. Youth employment and unemployment in July 2010, 2013 [cited 2013 Sep 15]. http://www.bls.gov/opub/ted/2010/ted_20100903.htm
